# Tilt-Pair Analysis of Images from a Range of Different Specimens in Single-Particle Electron Cryomicroscopy

**DOI:** 10.1016/j.jmb.2011.09.008

**Published:** 2011-11-11

**Authors:** Richard Henderson, Shaoxia Chen, James Z. Chen, Nikolaus Grigorieff, Lori A. Passmore, Luciano Ciccarelli, John L. Rubinstein, R. Anthony Crowther, Phoebe L. Stewart, Peter B. Rosenthal

**Affiliations:** 1 MRC Laboratory of Molecular Biology, Hills Road, Cambridge CB2 0QH, UK; 2 Rosenstiel Basic Medical Science Research Center, Howard Hughes Medical Institute, Brandeis University, Waltham, MA 02154, USA; 3 Max Planck Institute for Biophysics, D-60438 Frankfurt, Germany; 4 Molecular Structure and Function Program, The Hospital for Sick Children Research Institute, Toronto, ON, Canada M5G 1X8; 5 Department of Molecular Physiology and Biophysics, Vanderbilt University Medical Center, Nashville, TN 37232, USA; 6 Division of Physical Biochemistry, MRC National Institute for Medical Research, London NW7 1AA, UK

**Keywords:** EM, electron microscopy, 3D, three-dimensional, cryoEM, electron cryomicroscopy, TPPP, tilt-pair parameter plot, DLP, double-layered particle, DNA-PKcs, DNA-dependent protein kinase catalytic subunit, FAS, fatty acid synthetase, CAV, chicken anemia virus, PDH, pyruvate dehydrogenase, EMDB, Electron Microscopy Data Bank, electron microscopy, structure validation, particle orientation, beam-induced specimen motion, radiation damage

## Abstract

The comparison of a pair of electron microscope images recorded at different specimen tilt angles provides a powerful approach for evaluating the quality of images, image-processing procedures, or three-dimensional structures. Here, we analyze tilt-pair images recorded from a range of specimens with different symmetries and molecular masses and show how the analysis can produce valuable information not easily obtained otherwise. We show that the accuracy of orientation determination of individual single particles depends on molecular mass, as expected theoretically since the information in each particle image increases with molecular mass. The angular uncertainty is less than 1° for particles of high molecular mass (∼ 50 MDa), several degrees for particles in the range 1–5 MDa, and tens of degrees for particles below 1 MDa. Orientational uncertainty may be the major contributor to the effective temperature factor (*B*-factor) describing contrast loss and therefore the maximum resolution of a structure determination. We also made two unexpected observations. Single particles that are known to be flexible showed a wider spread in orientation accuracy, and the orientations of the largest particles examined changed by several degrees during typical low-dose exposures. Smaller particles presumably also reorient during the exposure; hence, specimen movement is a second major factor that limits resolution. Tilt pairs thus enable assessment of orientation accuracy, map quality, specimen motion, and conformational heterogeneity. A convincing tilt-pair parameter plot, where 60% of the particles show a single cluster around the expected tilt axis and tilt angle, provides confidence in a structure determined using electron cryomicroscopy.

## Introduction

Single-particle electron microscopy (EM), whether carried out on negatively stained or ice-embedded specimens, is growing in popularity and productivity as a result of steady technical improvements. Single-particle EM is one aspect of three-dimensional (3D) EM of biological macromolecules. It followed early applications of 3D reconstruction to helical,^1^ icosahedral,^2^ and two-dimensional crystalline arrays.^3^ The first reliable application of single-particle EM to particles with low symmetry was the negatively stained 50S ribosomal subunit.^4^ The potential impact of single-particle EM in structural biology was greatly expanded in the 1980s when Dubochet *et al.* developed their plunge-freeze method of embedding a solution of single particles in a thin film of vitreous ice.^5^ This simple procedure led to maps arising from images of the intrinsic molecular structure itself^6^ rather than the structure of a hollow shell of heavy-metal stain that outlined the surface contour of the macromolecule. The development of intermediate (∼ 300 keV) voltage microscopes with field emission electron guns further increased the quality of the images in the 1990s. Since then, following many near-atomic-resolution structures from two-dimensional crystalline or helical arrays,^7^ the resolution of maps from large unstained single particles in favorable cases has now reached near-atomic resolution,^8^ 3.3 Å in the best case,^9^  where it is possible to trace the path of the polypeptide backbone and assign side-chain densities.

With this increase in the range of specimens that can be studied and the pressure to extract the maximum amount of information from the images, the single-particle EM method is being pushed nearer to its theoretical limit, which can be defined by the minimum specimen molecular mass that allows unambiguous determination of the orientation parameters above the noise level in the residual for orientation determination.^10,11^ Clearly, this limit will depend on the nature of the specimen, such as shape, and the quality of the single-particle images. For example, DNA and RNA have higher contrast in ice than protein and suffer less radiation damage; thus, exposures with higher doses can be used for recording images. Images acquired on a detector with low detective quantum efficiency will contain less information than would be obtained with a perfect detector. Images that are blurred from beam-induced specimen motion or charging will contain less signal than those where specimen movement can be prevented,^12^ particularly at high resolution. It is hoped^13^ that these limitations will be overcome with much better images being recorded on nearly perfect detectors, so that single-particle EM can realize its full potential.

A typical electron cryomicroscopy (cryoEM) project involves the preparation of ice-embedded specimens, the subsequent recording of a number of low-dose micrographs, followed by picking a few thousand or a few tens of thousands of single-particle images from the micrographs. In a few cases, some projects have involved millions of particles.^14,15^ These images are then subjected to single-particle image analysis using one or more of a range of software packages, whose purpose is to sort out how the different views are related and calculate a 3D structure whose projections are consistent with the observed projection images after they have been corrected for the effects of the contrast transfer function (magnification, defocus, astigmatism, beam tilt, image drift/blurring). In favorable cases, where the structure is large and the images show clear, high-contrast features from the structure, any ambiguities or erroneously assigned orientations can be sorted out by iterative refinement using progressively more accurate 3D maps, and the procedure will converge on a single, correct overall structure, limited only by noise at high resolution. The procedure is thus a cyclical one in which the parameters that describe each single-particle image are varied with the goal of producing a single 3D map or, in some cases, a small number of maps that faithfully represent the structures whose projected images are observed in the original micrographs. However, the images are always noisy, being limited by the electron dose the structures can withstand before being irreversibly destroyed by radiation damage. As a result, the cyclical alignment is prone to producing orientations and a corresponding map that are trapped in a local minimum by the noise in the raw images.^16^ This bias becomes more serious for smaller structures or structures where the images display few or no strong, low-resolution features. In such cases, it is possible to end up with a 3D density distribution that has been derived from the initial images and represents a stable convergence of all the variable parameters yet is not a true representation of the structure being investigated. It represents either overrefined noise or a local minimum in parameter space from which the available software cannot escape. In many cases, the experimenter will suspect that the map is based on dubious orientation assignments and will use another procedure to obtain a more reliable starting model. For example, the random conical tilt procedure, demonstrated originally for negatively stained specimens,^17^ or the related orthogonal tilt method^18^ is often used to obtain an initial 3D map and has been used to produce 3D structures by cryoEM.^19,20^ Alternatively, electron cryotomography can be used, followed by sub-tomogram 3D averaging, in the hope of obtaining an unbiased 3D map. There are some very early examples of the use of tomography with negative stain^21,22^ and more recent ones involving cryoEM.^23,24^ However, even in these cases, the 3D maps sometimes have relatively low resolution and can be inadequate to produce reliable starting models for accurate refinement of the orientations of single-particle images. For example, a low-resolution structure may have no obvious features that determine the absolute hand, and this unresolved mirror symmetry may get locked in to attempts to extend resolution, in much the same way as it does in unrecognized twinned crystals in X-ray crystallography.^25,26^

How is it possible to prove whether a given structure is correct or not? This is a general problem that is most serious when the experimental map is near the current limits for molecular weight. Also, how do we know whether a structure is beyond the current limits and therefore likely to be wrong?

The idea that we develop further in this article is to check the consistency of the 3D map with the projections by recording pairs of images of the same single particles at different tilt angles. These images can then be used both to determine the absolute hand and to evaluate the image quality or the validity of the image-processing procedure by allowing an independent determination of whether the assignment of tilt angles and axes is correct. Since the publication of Rosenthal and Henderson,^27^ there have been very few published applications of the method. Apart from the five previously published studies that have been reexamined here,^27–31^ there have been only four others,^32–35^ which presented tilt analysis results of variable quality. We therefore thought that it was timely to carry out a systematic application of the procedure to a variety of large and small specimens with different symmetries, to demonstrate how easy the method is to apply and how informative the resulting tilt-pair parameter plots (TPPPs) are in analyzing the success of orientation determination. To our surprise, we also made some unanticipated observations that are intrinsically interesting and which suggest fruitful avenues for further investigation and potential improvements in the methodology.

## Results

### Large structures with high molecular mass

**Fig. 1:**
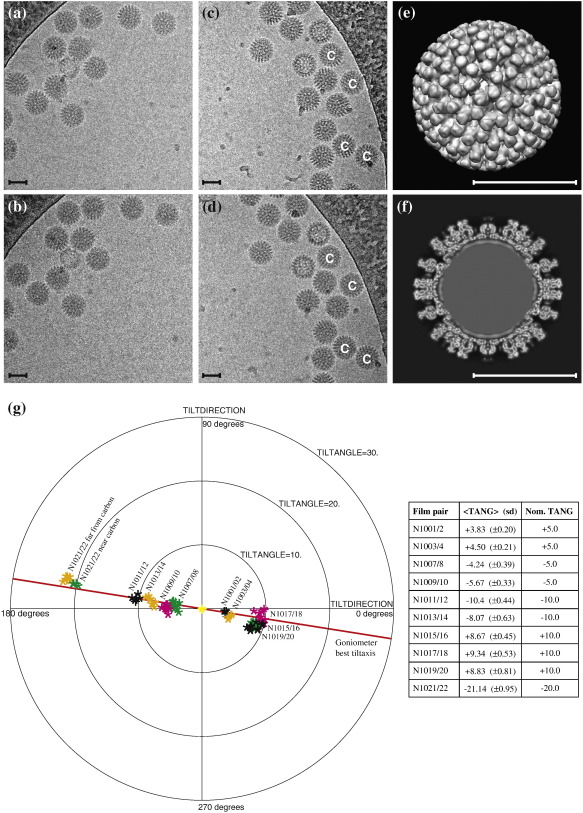
Rotavirus icosahedral DLPs (molecular mass, 50 MDa). (a and b) Untilted image N1017 and + 10° tilted image N1018, with relative tilt of + 10°. (c and d) + 10° tilted image N1021 and − 10° tilted image N1022, with relative tilt of − 20°. Note that images (b) and (d) were recorded after images (a) and (c) and show visible radiation damage. (e) Surface-shaded image of 3D map and (f) a section through the 3D map used to determine the orientation parameters. (g) TPPP for 10 tilt pairs recorded at different nominal tilt angles together with a table showing the experimentally determined and nominal relative tilt angles. The approximate standard deviation (SD) for the relative tilt angles, TANG, for each tilt pair is also given in parentheses. There were 95 virus particles in total, with the symbols representing each tilt pair being colored according to the micrograph from which it was selected. For the tilt pair N1021/22, the four particles that have TPPP values near the nominal, expected tilt are shown in a different color (green) in (g) and are labeled C in (c) and (d), because they are nearer the supporting carbon film. The red line in (g) shows the goniometer direction of tilt, which is at right angles to the tilt axis. Note that the outer radius of the plot has a 30° tilt angle. All scale bars represent 500 Å.

We started by examining a large well-determined structure to see how accurately we could determine the orientation parameters where the signal from the image of each single particle was substantial. We chose the rotavirus double-layered particles (DLPs) that Zhang *et al.* had shown could produce a 3D map at 3.8 Å resolution.^36^ These particles with icosahedral symmetry have a molecular mass of 50 MDa, with parts of the structure having *T* = 13 local or quasi-symmetry. In Fig. 1a and b and in Fig. 1c and d, we show typical tilt pairs of the rotavirus particles, each image being recorded with a dose of 20 electrons/Å^2^. Figure 1e and f show a surface-shaded representation and a central section, respectively, of the 3D map from Zhang *et al.* that was used to obtain the orientations.^36^ A series of 10 pairs of images tilted at different relative angles between − 20° and + 10° was recorded from 10 different areas of the grid. The orientations were determined by projection matching using the program FREALIGN^37,38^ and then used to plot the change in orientation between the two images using the program Tiltdiff.^27^ Tiltdiff shows the tilt angle and tilt axis required to rotate from one view of a 3D structure to another. The resulting TPPP is shown in Fig. 1g, where it is clear that the orientations of every virus particle on both the first and second image of each pair have been precisely determined. In fact, the projected images have such a strong signal that orientations almost as good as those plotted could be obtained using only the data from the images out to 35 Å resolution (see Fig. 6a), though the plotted data were obtained using information out to 15 Å. The inset table in Fig. 1g shows that the particles on each image pair are related by the same relative tilt axis and tilt angle, with a very small scattering around the center of each cluster. The pair with the tightest clustering is N1001/2, with an average scattering of 0.2°. The unanticipated observation was that the center of the cluster was not at the angle of + 5.0° set on the goniometer, but at + 3.8°, which is more than five standard deviations away from the nominal setting. The plot also shows clearly that the particles on each image pair are well clustered (e.g., N1001/2) but have tilt axes and tilt angles that are often well resolved from those from another tilt pair (e.g., N1003/4) recorded with identical goniometer settings. In some cases, such as for the pairs N1011/12 and N1013/14, the orientation changes between images do not even overlap. Not only do the measured relative tilt angles differ between pairs, but the azimuthal peak positions, representing the tilt axis direction, differ just as much (e.g., N1017/18, N1019/20), and it is clearly impossible for the goniometer tilt axis direction to vary at all, provided the imaging conditions are kept constant, which was the case. The only possible explanation is that the region of the ice-embedded specimen being imaged is moving during the exposure, presumably as a result of irradiation by the electron beam. It is also interesting that most of the particles in each tilt pair rotate in the same direction and by the same amount, so that it is the behavior of the local region of the thin film of ice that is being observed, not the behavior of individual particles. Different regions of the specimen move by different amounts and in different directions. The differences between the nominal and measured relative tilts can be in any direction and by any amount up to about 2°. The inescapable conclusion is that irradiation of an ice-embedded specimen with 20 electrons/Å^2^ at 300 keV causes the ice film to tilt by up to 2°, in a direction for which no pattern has yet been detected. For the particles in image pair N1021/22, there is a larger spread in behavior, with particles nearer to and farther from the surrounding carbon film in the field of view shown in Fig. 1c and d having over 1° difference in movement; hence, the ice appears to both tilt and bend. This observation, in which the orientation of the embedded icosahedral virus particles can be used to report and measure ice movement, is clearly interesting in itself. However, for the purposes of this article, we can conclude that the tilt-pair parameter plots prove that orientation parameters with precision to better than 0.2° can be obtained for these large structures, even when the particles themselves move by up to 2° during each exposure. Presumably, it is the average orientation of the particle as it moves during the exposure that is determined by projection matching.

### Small structures with low molecular mass

We next looked at some smaller structures, in which the signal from each particle is much smaller, roughly in proportion to molecular mass. We collected a complete set of single-particle data as well as some tilt pairs for the *Escherichia coli* enzyme β-galactosidase, which is a  450-kDa tetramer with *D*2 symmetry. We also reanalyzed the tilt-pair images from three earlier studies, using the improved approach described in Materials and Methods for determining tilt axes and tilt angles.

The improvement consists of carrying out a number of orientation searches in which the parameters used in the search, such as particle diameter, resolution, or defocus where there is uncertainty, are varied. The improved program, Tiltdiffmulti, then works out the best tilt axis and tilt angle relating the two views of a particle, by selecting the orientations from the different searches of the untilted and tilted views that best agree with the prior knowledge that the tilt axis must be nearly in the plane of the specimen. Note that even when the thin film of ice is itself moving slightly due to irradiation, the resulting net tilt (due to both the goniometer and the ice movement) is very likely to be about an axis close to the plane of the specimen, which would normally be within a few degrees of being at right angles to the beam. The use of Tiltdiffmulti with a small number (5 to 10) of independent runs of FREALIGN using slightly different parameters succeeds in increasing the number of successful tilt axis and tilt angle determinations, probably for particles where the correct orientation has a peak near the noise level.

**Fig. 2:**
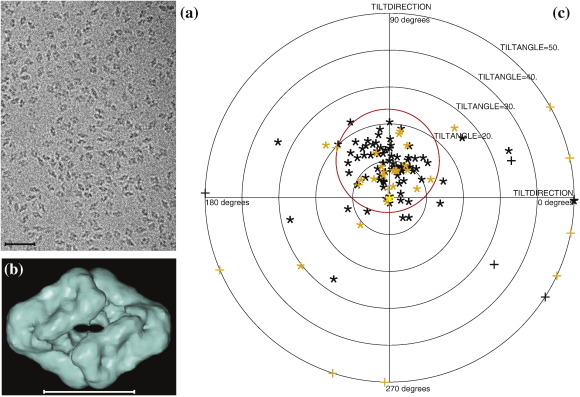
*E. coli* β-galactosidase with *D*2 symmetry and a molecular mass of 450 kDa. (a) Typical field of view (the scale bar represents 500 Å), (b) 3D map of β-galactosidase obtained using 6500 particle images processed using EMAN2 (the scale bar represents 100 Å), and (c) TPPP from particles from two image pairs (black and orange symbols), recorded with a relative tilt angle of 10°. The outer radius of the plot is 50°, the red circle has a radius of 14°, and it is centered at the expected tilt angle set on the goniometer.

Figure 2a shows a typical field of view of β-galactosidase particles. Figure 2b shows a surface-shaded representation of the 3D structure obtained using EMAN2,^39^ which will be reported in more detail elsewhere. Similar 3D maps from these β-galactosidase images have been obtained using a number of software packages such as IMAGIC^40^ and XMIPP.^41^ All the 3D maps look similar to a 3D map produced from the β-galactosidase atomic coordinates.^42,43^ Orientations determined using FREALIGN with any of these 3D maps produce a TPPP such as that shown in Fig. 2c, which shows the tilt parameters relating two pairs of images recorded at tilt angles of 0° and 10°. The particles from each image pair are plotted in a different color (orange or black). The cluster of points from both pairs is centered at 10° in one direction, as expected from the goniometer setting. A few of the particles (18) have tilts with errors that take them outside the 14°-radius circle on the plot and a few others (12, shown as “+” symbol) gave large out-of-plane errors in the tilt axis and angle determination, which occurs when the orientation of one or both members of the pair is wrong. However, out of 119 particle pairs, 72 (61%) have correct relative orientations within 10° and 101 (85%) have correct relative orientations within 25°, using the known goniometer settings as validation. This observation provides strong evidence that the 3D map being used for alignment and the orientations determined for the individual particle images are largely correct.

The tilt pairs from three published cryoEM structures of relatively small assemblies have been reanalyzed using the Tiltdiffmulti approach. All three had been successfully analyzed previously using another procedure developed by Rosenthal and Henderson.^27^ In this earlier procedure, the average phase residual of all the particle images from the tilted data was plotted over a range of tilt angles centered around the orientations obtained from the untilted image pairs and found to show a clear minimum at the correct tilt angle. This result proves that the 3D structures and the orientations of the particle images contain real information, but it is not quantitative in specifying what proportion of the orientations might be correct. Intuitively, however, the proportion of tilt angles that is correct is likely to be greater than 10–20%, since anything less would be unlikely to show a perceptible minimum.

**Fig. 3:**
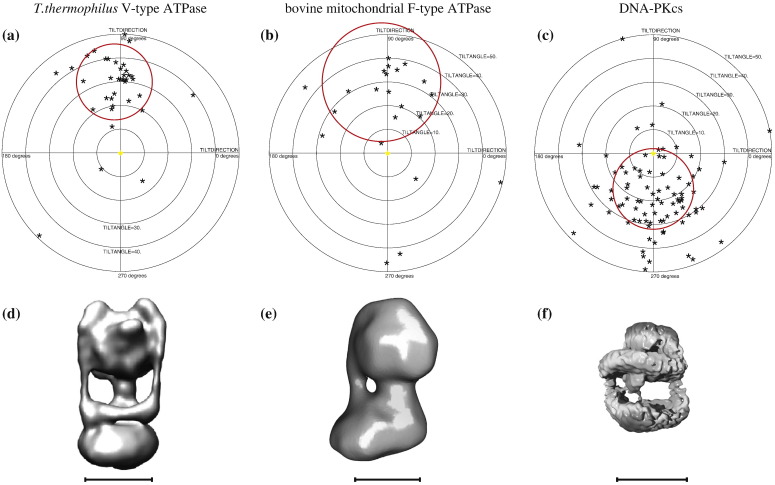
TPPPs for (a) V-type ATPase (molecular mass, 600 kDa), (b) F-type ATPase (molecular mass, 600 kDa), and (c) DNA-PKcs (molecular mass, 470 kDa), with surface-shaded representations of the 3D maps used for the orientation determination, (d) V-type ATPase, (e) F-type ATPase, and (f) DNA-PKcs. The radii of the red circles are 16°, 25°, and 17°, respectively, and the outer radii of the plots are 50°. The red circles are centered at the expected relative tilt angles of 30°, 30°, and 15°. These three structures have *C*1 point group symmetry (i.e., no symmetry). All scale bars represent 100 Å.

Figure 3a shows a TPPP for the individual particles from tilt pairs of *Thermus thermophilus* V-type ATPase, which has an overall molecular mass of 600 kDa and whose 3D surface-shaded structure is shown in Fig. 3d. The images and 3D map are from Lau and Rubinstein, in which the plot of the average residual on the tilted particles showed a highly significant phase difference of 14.9° between the structure with the correct hand and one with the opposite hand.^31^ As expected, the individual TPPP shows that most of the particle image pairs have reasonably well-determined orientations: 27 particle pairs (54%) have orientations correct to within 16°, with 72% within 40° and with only 20% having a substantial out-of-plane error in one of the images in the pair.

Figure 3b shows a similar plot for bovine mitochondrial F-type ATPase structure, which also has a molecular mass of 600 kDa with data taken from Rubinstein *et al.*^29^ As can be seen from the surface-shaded envelope shown in Fig. 3e, the F-type ATPase has less pronounced low-resolution features than the V-type ATPase, with only one stalk rather than two and no clear-cut collar. As a result, in the published plot of the average phase residual for the 29 particles, the phase difference between the minimum at the correct relative tilt angle and that in the opposite direction was only 9°. When we used the original orientation parameters, the TPPP from the individual particles did not show any clear clustering, but when we used the improved Tiltdiffmulti procedure, we obtained the TPPP shown in Fig. 3b, in which 15 out of 29 image pairs (52%) have good orientations within 25° and 65% within 60°. Although this is the lowest success rate for any of the tilt pairs in the structures we examined, it is consistent with the relatively low  30-Å resolution reported in the article.

Figure 3c and f shows the TPPP and the surface-shaded model of the DNA-dependent protein kinase catalytic subunit (DNA-PKcs; molecular mass, 470 kDa) from Williams *et al.*^30^ In the Supplementary Data that accompanied the DNA-PKcs publication, the plot of the average residual for the tilted particles showed only a 3° phase difference between the correct structure and one with the opposite hand; hence, there was always the worry that perhaps only a small proportion of the particle images had their orientations correctly determined. It is therefore satisfying to find in Fig. 3c that there is a clear and convincing clustering around the known 15° tilt angle used to record the second set of images for each pair. Out of the 108 particles used, the relative orientations of 47 particles (44%) are within 17° and those of 87 particles (81%) are within 50° of the known goniometer setting. We will discuss later what is the expected effect of errors in determining orientation on the resulting overall resolution of the final 3D map. For these smaller structures, however, it is comforting to know that the orientations determined in this way are largely in agreement with the specified goniometer angle settings, which were not known by the computer programs.

### Structures with intermediate molecular mass

Finally, we collected new tilt-pair images of *E. coli* 70S ribosomes and yeast fatty acid synthetase (FAS) and reanalyzed previously published tilt pairs from chicken anemia virus (CAV) and the catalytic domain of *Bacillus stearothermophilus* pyruvate dehydrogenase enzyme 2 (PDH-E2CD, or PDH). These are all structures of intermediate size, with molecular masses between 1.6 MDa and 2.7 MDa.

**Fig. 4:**
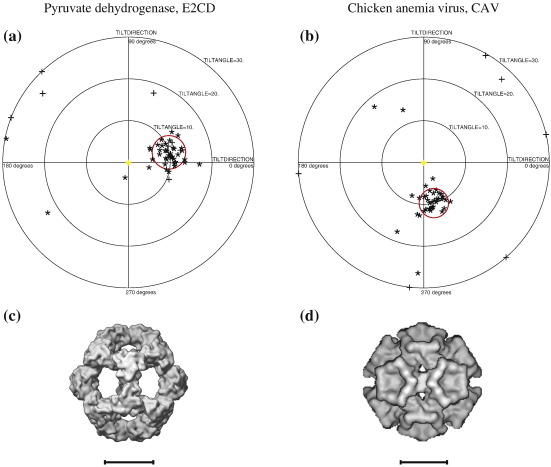
TPPPs for two icosahedral structures both with relative tilt angles of 10°. (a) PDH catalytic domain (molecular mass, 1.6 MDa); the red circle has a radius of 4°. (b) CAV (molecular mass, 2.7 MDa); the red circle has a radius of 3.5°. The surface-shaded 3D maps used for the orientation determination are shown in (c) PDH and (d) CAV. The data used for this figure are from Rosenthal and Henderson^27^ and Crowther *et al.*,^28^ respectively. The “+” symbols indicate particles whose out-of-plane error is greater than 1.5× the average. All scale bars represent 100 Å.

PDH has a molecular mass of 1.6 MDa. Since an extensive tilt-pair analysis was published previously,^27^ we simply replot the published data. Figure 4a and c is extracted from the earlier article. The TPPP shows that 31 particles out of 50 (62%) have angles that agree within 4° with the expected goniometer setting, with only 3 (6%) having large out-of-plane errors.

For CAV,^28^ a similar plot is obtained (Fig. 4b), although with slightly more accurate orientations since the structure is slightly larger at 2.7 MDa. The plot shows that 78% of the particles (35 out of 45) have reasonable orientations (within 10°), with 62% being within the 3.5°-radius circle in the plot.

**Fig. 5:**
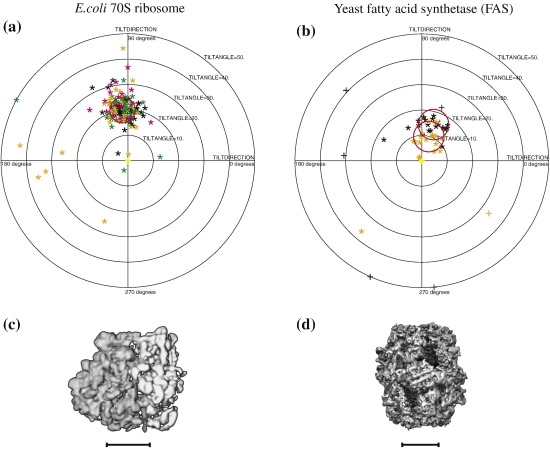
TPPPs. (a) *E. coli* 70S ribosome (symmetry, *C*1; molecular mass, 2.6 MDa). There were 12 tilt pairs recorded at − 10° and + 10° tilt angles to give relative tilt angles of 20°. The red circle has a 5° radius centered on the + 20° relative goniometer tilt. (b) Yeast FAS (symmetry, *D*3; molecular mass, 2.6 MDa). There were two tilt pairs with relative tilt angles of 10° (± 5°, orange symbols) and 15° (± 7.5°, black symbols). The red circles have radii of 6° centered on the expected positions. Surface-shaded views of the 3D maps used for the orientation determination are shown in (c) 70S ribosome and (d) FAS. The tilt-pair images used to produce these plots were collected as part of this work. The 3D maps were downloaded from the EMDB. All scale bars represent 100 Å.

For *E. coli* 70S ribosomes, a more extensive set of tilt-pair data with goniometer settings of − 10° and + 10° was recorded with 12 pairs of images containing 220 well-resolved particles. The orientations of the particles in both sets of images were obtained using FREALIGN to compare with either the 3D structure from Gabashvili *et al.*^44^ or an unpublished 3D structure obtained earlier using similar specimens as used for the tilt pairs. The Gabashvili map produced a slightly greater proportion of correct orientations but with a slightly larger scattering of angles. Figure 5a shows the resulting TPPP, which has different colors for particles from different images, and Fig. 5c shows the surface-shaded Gabashvili *et al.* structure that was used.^44^ Although the molecular mass of these empty ribosomes, at 2.6 MDa, is the same as that for CAV, the success rate in the TPPP analysis in terms of the angular scattering was lower, even though the images looked better in terms of higher contrast and less ice contamination. Only 45% of the particles (98) had orientations within the 5°-radius circle in the plot, compared with 62% within 3.5° for CAV. Similarly, 67% of the 70S ribosome tilt pairs had orientations within 10° of that expected, compared with 78% for CAV. Hence, there was a consistently greater spread in the angular clustering for ribosomes.

Lastly, for yeast FAS^45^ with a molecular mass of 2.6 MDa, Fig. 5b shows the results of TPPP analysis of two tilt pairs, one with angles of ± 5° and the other with angles of ± 7.5°, so that the expected positions of the particles in the plot should be at 10° and 15°, respectively. These are shown as orange and black symbols. The TPPP shows that 59% (26 out of 44 particles) have orientations within the 6°-radius circles and 82% within 15°. Thus, again, the clustering of the angles is significantly more diffuse than for either CAV or PDH.

We also asked whether there was better clustering for any particular tilt pair that could be correlated with defocus or ice thickness, since one would certainly expect some dependence on these parameters.^36^ However, since we had only one to two tilt pairs for most of the nine specimens as listed in Table 1, we could not detect any significant pattern. One of the FAS image pairs gave poorer clustering, but this was not due to ice thickness or defocus since those parameters were similar for all three FAS pairs.

## Discussion

The theory behind 3D structure determination or 3D reconstruction from a number of images of identical particles viewed in different orientations is rigorously based on Fourier analysis and the projection theorem.^1^ Provided the individual particles are identical and the image parameters (defocus, etc.), particle orientations (Euler angles), and translations have been correctly determined, the 3D structure that is calculated must be correct. Problems only arise if any of these conditions are not met, and a TPPP can provide reassurance that is objective and independent of the history of the structure determination.

### Validating 3D maps

The work reported here shows how reliably particle orientations can be obtained for a variety of specimens. The TPPP analysis provides an objective measurement of both the proportion of particles with well-determined orientation parameters and the accuracy of the individual parameters. A TPPP in which most of the particles are clustered around the tilt angle and tilt axis that is known only to the experimenter and not to the programs used to produce the plot shows conclusively that the Eulerian angles that describe the orientations of each image are related in a unique way to the 3D structure being used to identify the orientations. Although we have presented convincing TPPPs for nine different specimens, it is still too early to propose a simple validation formula to distinguish reliable from unreliable structures. In our experience, if less than 60% of the particles show a single cluster, the basis for the poor orientation determination should be investigated. If the orientation search and refinement cannot be further optimized, perhaps the current model is wrong or perhaps the particles need to be modeled by multiple structural species or structural heterogeneity.

The TPPP procedure provides a direct cross-validation of the orientation parameters but not for other key image parameters such as defocus or magnification. If the data used to produce the 3D map and to describe the pair of images of tilted particles are systematically wrong in the same way, the TPPP analysis could still work perfectly; thus, it is important that these other image parameters such as defocus and magnification are independently validated. There are many programs to perform this validation, such as CTFFIND3^46^ or EMAN2.^39^ Also, suppose that a particular structure is not composed of identical 3D molecules but consists of two or more domains with a flexible link: in this case, the orientation might lock onto one domain for the first image in the pair and to the other domain in the second, tilted image in the pair. The TPPP would then show a more pronounced scatter and possibly this is why the TPPPs for the empty ribosome and FAS are less precisely clustered than those for CAV and PDH, which have similar molecular masses.

It is also possible that a 3D map (determined by an independent method) that is being used to determine tilt angles might be mostly correct but have, for example, 20% of its mass positioned incorrectly. In such a case, the TPPP may still work well since the orientation determination would be dominated by the major part of the structure. This might then mislead the user into concluding that the structure was correct, whereas, in reality, part of it was wrong. However, provided a new model is calculated using angles determined in the same way as they were to produce the TPPP, it should be possible to remove this model bias. Hence, the use of TPPP can provide a useful cross-validation in cases of doubt, though clearly there are many published cryoEM single-particle structures that are so overdetermined that TPPP is unnecessary. Nevertheless, a TPPP provides reassurance and proof of the validity of the overall structure. Of course, at the other extreme, it might be more difficult to escape from the bias in an incorrect 3D map in which there is a more complicated mixture of the real structure with a twin, multiple twin, or mixed hand of the real structure, and a poor TPPP would be expected.

**Fig. 6:**
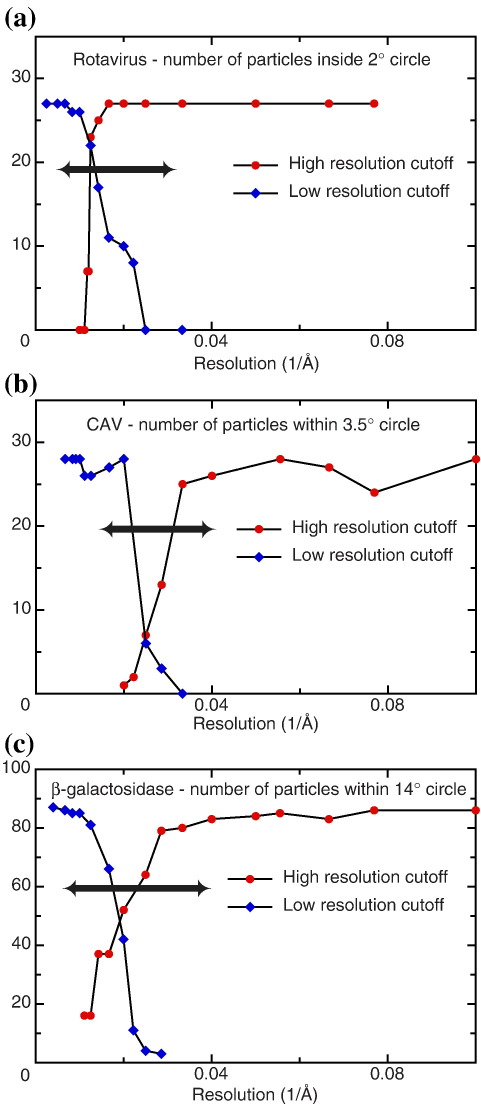
Number of particles in which the tilt-pair relative orientations are clustered around the expected tilt axis and tilt angle, plotted as a function of the lower- and higher-resolution cutoffs used in FREALIGN: (a) rotavirus within the 2°-radius circle, (b) CAV within 3.5°, and (c) β-galactosidase within 14°. The double arrowhead shows the resolution range that contributes most to the orientation determination. When the low-resolution cutoff was varied, the high-resolution cutoff was set to its maximum value, and vice versa.

A TPPP says nothing about resolution or about whether small, specific details of the structure are correct; it only says whether the overall 3D map is correct at the modest 15–20 Å resolution that is most important in orientation determination. The tests we have done, shown in Fig. 6, where the resolution of the Fourier components included in the TPPP analysis is varied, show that most of the orientation information in current images comes from Fourier components between 120 Å and 20 Å resolution, with much smaller contributions from outside this range. Therefore, it is clear that other resolution tests are still needed to measure what proportion of any higher-resolution information in a map is real and what proportion is overrefined noise.

A TPPP can differentiate between a good and a bad 3D map at lower resolution. For example, an initial low-resolution model, in which some of the particle images had been included with Euler angles that describe a structure with the opposite hand, will have an artificial extra center of inversion symmetry in the 3D map. This error will show up in the TPPP with some particles being clustered with a tilt angle that is the negative of the correct one. The plot will then show a less clear bias away from the origin, unlike all the TPPPs shown here, which show almost no particles with tilts in the wrong direction. Similarly, if one 3D map is noisier than another, but otherwise correct, the TPPP will show fewer particles with a broader clustering simply because the noise makes the orientation determination weaker. Although we have not presented data to show either of these situations for TPPP here, we have frequently observed both.

### Accuracy of orientation determination

Table 1 gives a comparison of the clustering of the orientations according to molecular mass. It shows clearly how the accuracy of the orientation determination is much better for large structures. Of course, this is expected because the amount of information in the image is proportional to the overall molecular mass, whereas the overall noise is proportional to the molecular area. Thus, signal-to-noise ratio should increase with size, though it is harder to be exact about the power law. The TPPP gives a quantitative estimate of orientation error, quite independently of other statistical criteria, such as the Fourier shell correlation.

By extrapolation, even for typical good-quality images such as those used here, there is also a minimum molecular weight below which most, and eventually all, of the orientations determined will be incorrect, thus badly affecting or even invalidating the quality and reliability of any structure. In Table 1, the angular error appears to be roughly proportional to the inverse of the molecular mass: the 100× bigger rotavirus has ∼ 60× less angular spread than the ∼ 0.5-MDa particles. If we then extrapolate the data in Table 1, it seems likely that, for molecular masses of half the size (250 kDa) of the four smaller structures examined here, the orientation error would be sufficiently large that the map would consist of a single, possibly elliptical, blob unless that structure had a distinctive shape or higher density than protein, such as for structures rich in nucleic acid. On the other hand, it is hoped that image quality will be improved once better detectors^47^ are available, better contrast images are obtained using a quarter-wave plate,^48^ or image blurring caused by beam-induced specimen movement is prevented or reduced.^12^

It should be noted that the clustering and accuracy of orientation determination described here represents a continuous error distribution and not a sampling, so that a ± 10° error in orienting a  150-Å-diameter particle would not simply result in a 25- Å-resolution structure as might be calculated from the formula relating resolution to angular sampling.^49^ We have tried to estimate the effect of a Gaussian distribution of angular errors on the effective resolution of a 3D map. An error in the angle of view is effectively a rotational blurring of the 3D object, giving rise to an additional temperature factor or *B*-factor that is included in the parameter *B*_computation_ that was described by Rosenthal and Henderson.^27^ The *B*-factor is a parameter that describes how contrast fades with resolution according to the definition *F* = *F*_0_·exp(− *B*/4*d*^2^). Thus, a *B*-factor of 400 Å^2^ causes structure factor amplitudes to fade to 36% at 10 Å or 1.8% at 5 Å. Table 2 presents an effort to develop an analytical relationship between angular uncertainty in the projection images and the increased *B*-factor of the resulting map. A high *B*-factor then leads directly to lower resolution, though a larger number of particles can be used to offset this to some extent. From the shading in Table 2 and the angular scatter in Table 1, it is clear that, at present, it is possible to determine orientations accurately enough to produce high-resolution maps only for larger particles. Small- and medium-sized particles produce low- and medium-resolution maps. Extrapolating again, a twofold improvement in angular accuracy for medium-sized structures (from 4° to 2°) or a threefold improvement for small structures (from 15° to 5°) should allow near-atomic-resolution maps to be obtained with reasonable numbers of particles. Hopefully, anticipated technological improvements will help to achieve these goals.

### Flexibility of 3D structure

We have noted that the scattering of the points for each particle in the TPPP seems to be greater for ribosomes and FAS than for CAV and PDH, even though the ribosome images show higher contrast because of the large RNA content. Since there is good evidence from many publications^50–52^ that ribosomes have a number of structural states such as those involved in “ratcheting” where parts of the structure can move by up to 10° or 12°, and FAS has structural flexibility as part of its enzymatic mechanism, it seems likely that their flexibility contributes to the increased scattering in the plots.

### Beam-induced translational and rotational motions

The observation of rotational motion of the rotavirus particles in Fig. 1 and the consequent blurring of the recorded images deserve further investigation to see whether imaging conditions that minimize the movement can be developed. However, it is worth noting that, for at least the rotavirus particles, the image blurring during each exposure will contribute more to an increased effective *B*-factor in the final 3D map calculated from similar particle images than any error in the orientation determination of each particle. In this case, *B*_image_^27^ will be greater than *B*_computation_,^27^ whereas the opposite will be true for the smaller- and medium-sized particles. How can images recorded with specimen movement of the type observed here possibly produce high-resolution structures, such as the 3.8-Å rotavirus structure?^36^ One possible explanation is that the movement consists of a tilt of the specimen about only one axis, so that the recording of the Fourier components in the other direction parallel with the tilt axis is unaffected. Although there will be a substantial blurring and reduction of power in one direction, the images will still contain some high-resolution information and the losses can be made up by symmetry averaging or simply by adding more data. Also, there may well be a fraction of particles that happens not to move much.

The observation of the behavior of ice films at liquid helium and liquid nitrogen temperatures during cryoEM^53^ and of frozen samples during X-ray crystallography^54^ suggests that an important consequence of radiation damage is CH bond breakage and subsequent release of hydrogen gas. The finding that lower dose rates can improve the dose at which bubbles of hydrogen gas are observed^55,56^ suggests that hydrogen gas production and the internal pressure it creates might be the cause of a substantial component of the specimen movement that we and others have observed. Thus, it may be possible to reduce the specimen movement and image blurring in cryoEM images by dose rate reductions.

### Hand (index of chiral power)

Clearly, determining the absolute hand of a structure is useful, and the TPPP allows this to be done in a very reliable way. Indeed, data of lower quality would still be quite adequate for hand determination.

If a map is not good enough to differentiate between itself and its mirror image, then perhaps it has too little information to be informative and is therefore not worth publishing. This might show up in the TPPP by showing a scattering of the particle parameters, with half of them centered around a positive tilt angle and axis and the other half centered about an equivalent negative tilt angle. If in addition there is a broad scattering in the TPPP, then the plots will not show any clear relative orientation at all. For example, in Fig. 6a from Rosenthal and Henderson,^27^ in which the 3D map and the orientation search parameters were poor, there was no clear clustering and the map had a lower resolution and an ambiguous hand.

A structure like this may have been calculated from images that contained too little information to determine the orientations unambiguously; hence, the initial structure may have been a mixture of structures, related to one another by rotations or inversions in much the same way as the molecules in a merohedrally or tetartohedrally twinned 3D crystal. Such an initially incorrect model could then easily become locked in, generating model bias from which it cannot escape. This might happen if the overrefined noise from each image has a greater power than any differences between the structure and its mirror image or rotationally degenerate “twin”. An index of chiral reliability that consists of the ratio of mean density difference between a structure and its mirror image (after finding the best rotational superposition) and the mean noise level might be useful.

### Radiation damage and electron dose

There is one important reservation about the use of successive images. In recording more than one image from the same specimen area, whether tilted or not, the second and subsequent images will show a greater amount of radiation damage. Many studies have shown that the high-resolution Fourier components are destroyed more rapidly than those at lower resolution;^57,58^ thus, it is likely that the signal-to-noise ratio in the second image will be lower, making the orientation determination likely to be less successful and less accurate. As a result, the plots shown in the figures may give a slightly pessimistic view of the accuracy of the orientation determination of the first image. Even if there was no radiation damage and both images had the same signal-to-noise ratio and the same orientation accuracy, the plots would be expected to have a √2 increased scatter compared with the individual error on a single image (assuming the orientation errors are uncorrelated); thus, it would still be desirable to develop better methods of estimating the accuracy of the orientation determination.

### Lessons for future data collection strategies

It is interesting that the use of the program Tiltdiffmulti to select the best pair of orientations from those produced by a number of runs of the program FREALIGN gives a higher success rate than the use of a pair of Euler angles from a single run. The ability to detect a more reliable tilt angle in this way is due to the introduction of the additional powerful constraint that the tilt axis is in the plane perpendicular to the viewing direction. This constraint can be applied to tilt pairs of images without specifying the direction of the tilt axis or the magnitude of the tilt angle. It may therefore be that determination of particle orientation using tilt pairs rather than individual images will help to extend the cryoEM method down to single particles of smaller molecular weight, even if only the first image is included in the final 3D map. In principle, a constraint based on knowledge of the tilt axis and tilt angle values could be used to improve orientation determination, after an initial data set is found to have a convincing TPPP. Although such a data collection and processing strategy is more complicated, the procedure could be automated.

### Improvements with better images and better detectors

Although the results presented here imply that the resolution and reliability of cryoEM are intrinsically limited due to the restricted information in the particle images, this does not take into account the improvements to the images that may be made in the near future. It is certainly expected that the improved detective quantum efficiency obtainable with back-thinned CMOS detectors at high (300 keV) energy,^59,60^ which are just becoming available commercially, will improve the resolution and signal-to-noise ratio in the raw images. If quarter-wave plates^48^ and Cc correctors^61^ can also be made more robust and affordable, and if the specimen movement, consisting of both translational and rotational motions induced by irradiation, can be eliminated or reduced, then the future of single-particle cryoEM and the possibility of atomic-resolution structures for single particles as small as 100 kDa seem assured.

## Materials and Methods

### Overview

We have examined nine different specimens, all of which produced convincing TPPPs. The data used here for the analysis of β-galactosidase were entirely new, consisting of a complete single-particle EM data set, the resulting 3D map, and the tilt pairs. For three other specimens, namely, rotavirus DLPs, *E. coli* 70S ribosomes, and yeast FAS, published 3D maps were used together with new tilt pairs collected from fresh specimens. For another four specimens, namely, the two ATPases, DNA-PKcs and CAV, published data including previously recorded tilt pairs were reanalyzed using new programs and algorithms. Finally, for PDH, the type of analysis described here had been done previously^27^ and is thus simply replotted for comparison.

### 3D maps

The provenance of the 3D maps used here is listed in Table 3. The maps for eight of the specimens were from previously published work. In three cases, these 3D maps could be obtained from the Electron Microscopy Data Bank (EMDB) and are listed by their access numbers. In the other cases, the maps were obtained from the authors of the publication listed in the table or from one of the authors of this article. It is intended that all the 3D maps used here (and the tilt-pair stacks) will be deposited in the EMDB; hence, they will be more generally available. In the case of *E. coli* β-galactosidase, a new 3D map had to be calculated. The 3D map of β-galactosidase was obtained in two ways. First, a series of 32 micrographs was recorded from specimens of β-galactosidase obtained from Sigma (catalog no. G3153). Briefly, solutions of β-galactosidase at a concentration of 1 mg/ml were applied to glow-discharged Quantifoil grids (Agar Scientific), blotted, and plunge frozen using a homemade apparatus similar to that described by Dubochet *et al.*^5^ Grids were transferred to an FEI Polara G2 microscope and images were recorded on film at 39,000× magnification and 80 keV with defocus between 1.5 μm and 2.0 μm using an electron dose of  8 electrons/Å^2^ and developed for 12 min in full-strength D19 developer. The micrographs were digitized on the KZA film scanner^62^ in  6-μm steps, defocus was estimated using CTFFIND3,^46^ and particles were picked manually using Ximdisp^63^ and then processed using EMAN2^39^ to give a structure at ∼ 13 Å resolution, which will be described in more detail in a later publication. We also calculated a 3D map starting with the atomic coordinates (Protein Data Bank: 3I3E) of *E. coli* β-galactosidase.^43^ These coordinates were rotated and translated, using a purpose-written program called D2rottran, to be centered at an origin of (0,0,0) in a 300 Å × 300 Å × 300 Å cubic unit cell, with the *D*2 particle symmetry axes parallel with the cell axes, and used to calculate a 3D map. An approximate solvent correction was made by subtracting a 3D map calculated using the CCP4 program FFT (Fast Fourier Transform) with a *B*-factor of 2000 from a 3D map with a *B*-factor of 400 in a proportion of 4:5. We thus ended up with a map whose low-resolution features were not overemphasized by the *in vacuo* coordinates and that closely resembled the experimental map obtained using EMAN2, by having also a *B*-factor of 400, which we estimated to be the *B*-factor of the processed image data.

The surface-rendered representations of the nine different structures shown in the figures were reproduced from the original publications or for β-galactosidase made within EMAN2^39^ or for FAS using Chimera.^64^

### Tilt-pair images

Tilt-pair images were obtained by cryoEM from four specimens.

For β-galactosidase, these were recorded with a tilt angle of 10° using the same grids as described above on an FEI Krios at a magnification of 150,000× and at 80 keV, using an electron dose of  15 electrons/Å^2^ on an FEI Eagle 4K × 4K CCD detector. The images were binned 3 × 3 to give a pixel size of 3.0 Å, and 119 particles were picked from two image pairs.

For rotavirus DLPs, cryoEM specimens were prepared as before^36^ but using Quantifoil (R 1.2/1.3) grids washed with ethyl acetate before glow-discharging. Ten tilt pairs were recorded on a Gatan US1000 2K × 2K CCD detector, on an FEI F30 microscope at 50,500× magnification and 300 keV, at a dose of  20 electrons/Å^2^, using tilt angles from − 20° to + 10° as listed in Fig. 1. After picking the particle pairs manually, the stack of images was binned 2 × 2 to give a pixel size of 5.9 Å.

Empty *E. coli* 70S ribosomes were kindly provided by Ann Kelley, prepared according to Milon *et al.*^65^ Grids for cryoEM were prepared by applying 70S ribosomes at 50 nM concentration (∼ 0.2 mg/ml) to glow-discharged Quantifoil grids using an FEI Vitrobot. Grids were then transferred to an FEI Krios cryomicroscope, and images were recorded with tilt angles of ± 10° at a magnification of 84,500× and at 200 keV on an FEI Eagle 4K × 4K CCD detector using a dose of  15 electrons/Å^2^. Particles were picked manually from 12 image pairs and binned 3 × 3 to give a pixel size of 5.3 Å.

For yeast FAS, grids were prepared following the procedure described by Gipson *et al.*^45^ and transferred to an FEI Polara G2 and image pairs at relative tilt angles of 10° or 15° (± 5° and ± 10°) recorded at 200 keV and at a magnification of 80,700× on a Gatan US4000 4K × 4K CCD detector using a dose of  12 electrons/Å^2^. After picking 91 particles from three tilt pairs, the images were binned 2 × 2, to give a pixel size of 3.7 Å. After some preliminary analysis, the two best tilt pairs with 44 particles were selected for more extensive processing.

### TPPP procedure using Tiltdiff or Tiltdiffmulti

TPPP, produced by the program Tiltdiff,^27^ shows the tilt angle and tilt axis required to rotate from one view of a 3D structure to another. The input to Tiltdiff^27^ consists of two lists of Euler angles (φ, θ, ψ) that describe the rotations of the 3D map, using the ZYZ Euler angle convention, which are required to give the projection images seen in the untilted and tilted views of the specimen. In the work described here, we use the program FREALIGN on all nine specimens to search and refine these orientation parameters. The program Tiltdiff then searches for the tilt axis and tilt angle that best relate the Euler angles from the two views, restricting the search only to tilt axes that are in the plane of the specimen and calculating a residual error in the position of the best fit obtained that is roughly equivalent to an out-of-plane error. Since the experimenter knows that the actual tilt axis must be in the specimen plane, there is an option either to plot or to omit the particles whose out-of-plane error exceeds a certain angle, in this case greater than 1.5× the average out-of-plane error. If the particles are plotted, they are indicated by a “+” symbol rather than an “⁎”. Tiltdiff and Tiltdiffmulti take the particle symmetry into account to ensure that the tilt angle and tilt axis are not affected by the rotational degeneracy of the particle.

During the course of the work, it was noticed from time to time that, if the FREALIGN orientation searches were carried out using different parameters such as particle outer radius, magnification, resolution cutoff, or defocus, the Tiltdiff plots would also differ in a very interesting way. For some particles, the most successful tilt angle determinations, judged by how close they were to the known tilt angle and tilt axis, would occur with different search parameters than for other particles. This could be for such an obvious reason that the end-on view of an asymmetric particle, such as β-galactosidase, occupies a smaller area so that a tighter mask obtained by using a smaller particle outer radius limits the image area to the area with the signal. It could also be that a defocus gradient existed across the field of view of particles and therefore that some particles had a different defocus from others. However, the possible principal cause is that some of the correct particle orientations had residuals that were close to the noise level and that small changes in the data, such as for example by slightly varying the resolution cutoff, would make the correct orientation appear to be above or below a strong noise feature in the angular search. An improved program, Tiltdiffmulti, which allowed a list of several Euler angles from the untilted particle to be compared with a similar list from the tilted particle, was therefore devised. The program then chooses the orientations that agree best with the prior knowledge that the tilt axis must be in the plane of the specimen. Apart from the additional power due to considering more than one possible orientation, Tiltdiff and Tiltdiffmulti are otherwise the same. This produced a significant increase in the number of particles in which the relative orientations of the tilt pairs were close to the values the experimenter had set on the microscope goniometer and therefore likely to be correct. It should be emphasized that the procedure, consisting of running FREALIGN several times on the tilted and untilted data followed by Tiltdiffmulti, has no knowledge of either the magnitude of the tilt angle or the direction of the tilt axis; hence, the clustering of most of the particles around the known relative orientation proves the validity of the angle determination. No fortuitous clustering was ever observed other than near the correct position. Although we have used only the program FREALIGN to determine the Euler angles, there is no reason that any of the other available EM software packages could not be used instead, provided the same ZYZ Euler angle convention is used.

In practice, there were a number of critical practical steps to ensure a good outcome. First, the quality of the specimens and the images must be good. This means that the thickness of the ice layer should be as thin as possible. There should be minimal contamination of the specimen. There should be no visible drift or charging. There should be visible Thon rings^66^ in the computed FFT of the images, and the defocus and astigmatism values obtained, for example by using CTFFIND3, should be consistent within the series of micrographs.

Second, if the tilt pairs were recorded at a different time or on a different microscope from the images used to produce the 3D structure, then the magnification is likely to be different. We have therefore found it essential to carry out a preliminary search (from a series of FREALIGN runs) over a range of magnifications from 0.9× to 1.1× of that expected, followed by plotting the phase residual. If everything else (defocus, pixel size, etc.) has been correctly determined, there will be a pronounced minimum in the phase residual that allows the relative magnification of the tilt pairs to be determined within 0.5%. If not, then there is usually something wrong with one of the other parameters. At this stage, it is often worth testing out a few different resolution ranges for the calculation of phase residual. For most of the specimens, a starting range of 150 Å to 18 Å seemed to work well and should give average phase residuals between 55° and 65°. This means that we have not used any high-resolution information to determine orientations. A residual higher than 65° probably means that the resolution used in the search should be reduced. Another key parameter is the maximum particle diameter RO in FREALIGN. This should be chosen to exclude noise from the area of the image around the particle. Some particles are asymmetric; a small radius may be appropriate for some views, and for others, a larger radius may be appropriate. Allowing the radius to vary slightly around the largest particle dimension may produce better orientations that can then be selected by Tiltdiffmulti.

Finally, once each TPPP has been produced, it is useful for display purposes to plot a circle (shown in red on all the plots) that shows how well the individual particle orientations are clustered. The radius of this circle can be selected to show whatever the experimenter wants. In this article, we have tried to draw it so that about half (range, 40–60%) of the relative particle orientations are within the circle. The red circles are centered at the position expected from the goniometer setting. The percentages within the circles in Figs. 2–5 are shown as the first number in the “Successful alignment” column in Table 1.

### Formula relating *B*-factor to orientation error

The formula used to produce the data in Table 2 relates an angular orientation error for a particle of a given size to an apparent *B*-factor due to the blurring of the density in the resulting map. It was derived as follows.

The function *f*(*x*) = exp(− 4*x*^2^/*X*^2^) is a Gaussian of full width *X*. It falls to a value of 1/*e* at a distance *X*/2 from the origin. Its Fourier transform, being proportional to exp(− π^2^*X*^2^/4*d*^2^), where *d* is the spacing in reciprocal space, has a *B*-factor of π^2^*X*^2^.

The mean square blurring due to an angular error of Δθ for a structure of diameter *D* = 2*R* is given by *X*^2^ = _0_∫^R^4π*r*^2^*dr*·*r*^2^Δθ^2^/[4/3·π*R*^3^] = 3/5·*R*^2^Δθ^2^ = [3/20]·*D*^2^Δθ^2^, when Δθ is in radians.

Hence, *B* = π^2^*X*^2^ = π^2^·[3/20]·*D*^2^Δθ^2^·[π^2^/180^2^] = (*D*·Δθ)^2^/2200, when Δθ is in degrees.

## Author Contributions

J.Z.C. and N.G. contributed the data for Fig. 1; S.C., those for Fig. 2; J.L.R., those for Fig. 3a and b; P.L.S., those for Fig. 3c; P.B.R., those for Fig. 4a; R.A.C., those for Fig. 4b; L.A.P., those for Fig. 5a; and L.C., those for Fig. 5b (as described in Materials and Methods). R.H. organized the collaboration, carried out the tilt-pair analysis, and drafted the paper. Everyone contributed to the final paper..

## Figures and Tables

**Table 3:** Source of 3D maps and tilt-pair images

Specimen	3D map	Tilt-pair images	Reference
Rotavirus DLP	dlp_ccd.mrc	This work, 95 particles	36
CAV	cav_pad2k.map	1 image pair 6216/6217, 45 particles	28
70S ribosomes	emd_1003.map	This work, 220 particles	44
FAS	emd_1623.map	This work, 44 particles	45
PDH-E2CD	pdh3d2k_cent.map	1 image pair 1982/1983, 50 particles	27
Thermus V-ATPase	model_128x128_6A.mrc, emd_1888.map	1 image pair, 45 particles	31
Bovine F-ATPase	final_small.mrc	1 image pair, 29 particles	29
DNA-PKcs	threed_map.mrc	108 particles selected from 191 particle pairs	30
β-Galactosidase	this work, 3i3e_rottran.map	This work, 119 particles	—

**Table 1:** Overview of tilt-pair statistics

Specimen	Symmetry	Particle size (Å)	Molecular mass (MDa)	Number of tilt pairs	Number of particles	Successful alignment (%)	Angular error (°)	
							Mean	Maximum
Rotavirus DLP	*I*2	700	50	10	95	100/100	0.25	1.0
CAV	*I*2	255	2.7	1	45	62/82	2.5	3.5
70S ribosomes	*C*1	270 × 260	2.6	12	220	45/75	4.0	5.0
FAS	*D*3	260 × 220	2.6	2	44	59/95	4.0	6.0
PDH-E2CD	*I*1	280	1.6	1	50	62/94	3.0	4.0
*Thermus* V-ATPase	*C*1	250 × 140	0.6	1	50	54/80	10.0	16.0
Bovine F-ATPase	*C*1	250 × 140	0.6	1	29	52/79	20.0	25.0
DNA-PKcs	*C*1	150 × 120	0.47	14	108	44/81	15.0	17.0
β-Galactosidase	*D*2	180 × 130 × 95	0.45	2	119	74/91	10.0	14.0

The column labeled “symmetry” gives the particle point-group symmetry where *I*1 and *I*2 refer to the two icosahedral axis conventions: *I*1 is the convention used in the International Tables with a fivefold along the direction (01*t*) where *t* = (1 + √5)/2, whereas *I*2 is that defined earlier by Crowther *et al.*,^2^ with a fivefold along (10*t*). In the column labeled “particle size”, one number is given if the particle is roughly spherical, two numbers when the particle is roughly in the shape of a tall cylinder, and three numbers when the particle is lozenge shaped. In the column labeled “successful alignment”, the first number describes the percentage of particles whose tilt axis/tilt angle are within a circle whose radius in degrees is given by the number in the last column. The red circles in Figs. 2 to 5 are also drawn to represent this same angular error. The second number describes the percentage of particles whose tilt axis is in the plane of the specimen again to within the same angular error. The penultimate column gives an estimate of the average angular orientation error derived from the clustering in the TPPP and is slightly smaller than the angle in the final column used to define the overall percentage of success.

**Table 2:**
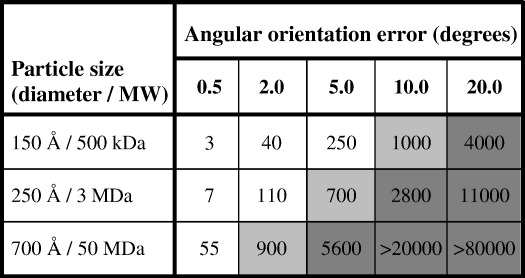
Effect of angular accuracy on *B*_computation_ and map resolution

Angular error is translated into an apparent *B*-factor due to computational blurring of the 3D map, using the formula *B* = (Δθ·*D*)^2^/2200, where Δθ is the orientation error in degrees and *D* is the particle diameter in Ångstroms. In previously published work, *B*-factors of *B* = 1000 have given 8.7-Å-resolution maps,^27^*B* = 750 gave 7.0 Å resolution,^3^ and *B* = 240 gave 3.3 Å resolution,^9^ though other factors including the number of particles in the data set are also important. This suggests that a two- to threefold improvement in orientation accuracy would allow structures of around 500 kDa to reach near-atomic (∼ 4 Å) resolution, without too many particles being required. The box shadings (white, pale gray, and dark gray) represent the likelihood of obtaining high-resolution (3–5 Å), medium-resolution (6–10 Å), or low-resolution (below 12 Å) maps with the given error in orientation angles.
